# Geriatrics-Focused vs Traditional Primary Care in the Veterans Affairs Health Care System

**DOI:** 10.1001/jamanetworkopen.2024.54865

**Published:** 2025-01-16

**Authors:** Susan N. Hastings, Courtney H. Van Houtven, Catherine L. Stanwyck, Chelsea R. Perfect, Justine Seidenfeld, Karen M. Stechuchak, Eleanor McConnell, Brenda L. Plassman, Kaileigh G. Byrd, Chelsea L. Whitfield, Valerie A. Smith

**Affiliations:** 1 Center of Innovation to Accelerate Discovery and Practice Transformation, Durham Veterans Affairs (VA) Health Care System, Durham, North Carolina; 2 Department of Medicine, Duke University School of Medicine, Durham, North Carolina; 3 Department of Population Health Sciences, Duke University School of Medicine, Durham, North Carolina; 4 Center for the Study of Aging and Human Development, Duke University School of Medicine, Durham, North Carolina; 5 Geriatrics Research, Education, and Clinical Center, Durham VA Health Care System, Durham, North Carolina; 6 Duke-Margolis Center for Health Policy, Durham, North Carolina; 7 Department of Emergency Medicine, Durham VA Health Care System, Durham, North Carolina; 8 Department of Emergency Medicine, Duke University School of Medicine, Durham, North Carolina; 9 Duke University School of Nursing, Durham, North Carolina; 10 Department of Psychiatry and Behavioral Sciences, Duke University School of Medicine, Durham, North Carolina; 11 Department of General Internal Medicine, Duke University, Durham, North Carolina; 12 Department of Biostatistics and Bioinformatics, Duke University School of Medicine, Durham, North Carolina

## Abstract

**Importance:**

Older adults often require specialized health care expertise, but the effects of geriatrics-focused models of primary care have not been fully evaluated.

**Objective:**

To compare the effects of geriatrics-focused primary care vs traditional primary care for older patients in the Veterans Affairs (VA) health care system.

**Design, Setting, and Participants:**

In this cohort study, geriatrics-focused primary care and traditional primary care patient dyads matched on variables associated with geriatrics-focused primary care entry and outcomes were enrolled from VA medical centers with operational geriatrics-focused primary care clinics serving 500 or more patients annually in fiscal year 2016. Across 57 eligible sites, patients 65 years or older who transferred their care to geriatrics-focused primary care within the previous 12 months (exposure period) (n = 291) and their matched traditional primary care controls (n = 277) were enrolled. Study recruitment occurred from March 22, 2018, through December 11, 2019, with patient outcomes data collection completed June 24, 2021. Data were analyzed from June 2023 to January 2024.

**Exposure:**

At least 2 geriatrics-focused primary care visits during the exposure period. Geriatrics-focused primary care involves interprofessional teams of clinicians with geriatrics expertise to provide comprehensive primary care for older veterans with complex needs.

**Main Outcomes and Measures:**

The primary outcome was home time (days not in a hospital unit, short-term nursing facility, inpatient rehabilitation, or an emergency visit) within 18 months of the exposure period. Secondary outcomes were completion of advance directives, assessments (falls, incontinence, and function), and medication quality (potentially inappropriate medications, Anticholinergic Drug Scale score). Patients were surveyed over 18 months about care experiences using Patient Perceptions of Integrated Care subscales, and Patient-Reported Outcomes Measurement System self-reported health and well-being measures.

**Results:**

A total of 568 patients were enrolled (mean [SD] age, 80.5 [7.0] years; 558 [98.2%] male), including 291 in the geriatrics-focused primary care and 277 matched in the traditional primary care groups; most baseline variables were comparable between the 2 groups. Geriatrics-focused primary care was not associated with more home time (rate ratio, 0.97; 95% CI, 0.62-1.51) over 18 months. Geriatrics-focused primary care was associated with higher odds of assessment for falls (odds ratio [OR], 2.86; 95% CI, 1.62-5.04), incontinence (OR, 2.73; 95% CI, 1.69-4.42), and function (OR, 4.05; 95% CI, 2.39-6.85) at 18 months. Geriatrics-focused primary care was not associated with advance directive completion, medication quality, or self-reported health and well-being.

**Conclusions and Relevance:**

In this cohort study, geriatrics-focused primary care did not increase time at home or other measures of self-reported health and well-being or integration of care within the first 18 months after care transition. However, increases in some process measures important for quality of care were observed, suggesting that further studies are needed to evaluate outcomes over a longer time frame.

## Key Points

**Question:**

What is the comparative effectiveness of geriatrics-focused vs traditional primary care on quality of care and patient experience for older veterans receiving care in the Veterans Affairs health care system?

**Findings:**

In this cohort study of 568 older veterans, transitioning care from traditional to geriatrics-focused primary care was not associated with a difference in home time over 18 months. Transitioning care was associated with statistically significant higher odds of receiving falls screening, incontinence screening, and functional assessments.

**Meaning:**

These findings suggest that further studies are needed to evaluate outcomes over a longer time frame.

## Teaser

This cohort study assesses the association of geriatrics-focused vs traditional primary care with quality of care and patient experience of care among older adult patients in the Veterans Affairs health care system.

## Introduction

Older adults with complex care needs represent a large and rapidly growing population of veteran patients in the Department of Veterans Affairs health care system (VA). In 2021, over 10 million veterans were 65 years or older. This growth in the older veteran population mirrors that of the US population overall. With shortages of geriatricians expected to continue,^1^ health systems today are investing in geriatric training for multiple specialties across inpatient and outpatient settings, including primary care, to meet the needs of older adults with medically complex needs.^2^ Increasing geriatric skills in the primary care workforce could improve clinical outcomes and patient experiences among older adults, given that geriatrics uses interprofessional team-based care and focuses on patient preferences in the care encounter.^3^

The VA has long delivered primary care using a patient-centered medical home model referred to as a PACT (patient-aligned care team).^4^ Team-based primary care homes allow for coordinated care aimed at prevention of acute illnesses and chronic disease management for a panel size of approximately 1200 individuals.^4,5,6^ Benefits of patient-centered medical homes for primary care have been well-established, including in the VA^7^; however, it is unclear how well primary care medical homes meet the needs of older adults with complex needs. The VA has more recently developed a special population PACT, GeriPACT, which was designed to integrate and coordinate traditional ambulatory health care services with a variety of community-based services by providing primary care with a focus on quality of life, complex psychosocial environment, and multimorbidity for a particularly vulnerable—and often frail—population.^8^ Although GeriPACTs mirror PACTs in that they act as a veteran’s patient-centered medical home within the VA health system, they differ from PACTs in several key aspects: GeriPACT clinician panel size is two-thirds that of a traditional PACT; receipt of geriatrics-focused training for team members; and the requirement of interdisciplinary health care professionals.^8,9^ Despite these known differences in the structure of the GeriPACT care model, we lack essential information about how GeriPACTs differ from traditional PACTs in terms of clinical quality measures that are relevant for older adults. We also lack an understanding of patient experience of care from the perspectives of the people who matter the most: older adult patients.

The overarching objective of this study was to compare the association of GeriPACT vs PACT with quality of care and patient experience of care among older adults. In addition to using electronic health records (EHRs) for quality-of-care outcomes, we enrolled older patients and longitudinally followed them up for 18 months to understand their initial experiences in geriatrics-focused primary care. Understanding how quality and experience of care differ when a patient moves from usual care (in PACT) to GeriPACT can help inform decisions about investment in geriatrics-focused primary care nationally by illuminating the outcomes of such care compared with usual primary care.

## Methods

### Ethical Declaration

This study was approved by the Institutional Review Board of the Durham VA Health Care System, Durham, North Carolina, and all participants provided verbal informed consent. Participants were compensated up to $80 for completing all surveys. Reporting was guided by the Strengthening the Reporting of Observational Studies in Epidemiology (STROBE) guideline.

### Study Population and Treatment Assignment

This study focused on GeriPACTs with similar features by selecting participants from sites with fully implemented GeriPACT clinics (≥500 patients annually; ≤800 patients per team; a social worker and a pharmacist included on team). Patient-level eligibility criteria (see eMethods in Supplement 1) were assessed via medical record review and phone screening. Briefly, inclusion criteria were 65 years or older and a valid phone number in the EHR; exclusion criteria included inability to communicate by phone, active substance use or psychosis, lack of decision-making capacity without a proxy, hospice or palliative referral with a life expectancy of less than 6 months, or current institutional care.

**Figure 1.**
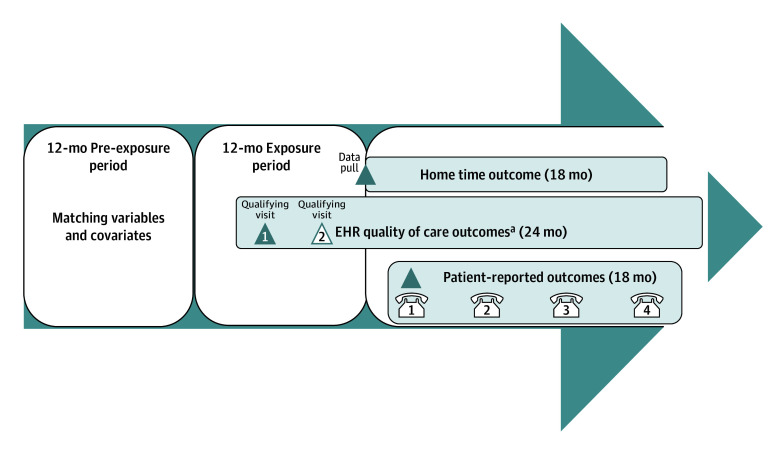
Study Flow Timeline Triangles indicate time 0 for each set of analyses. Telephone icons indicate phone contact. Study consent was obtained at phone call 1. ^a^Includes screening, advance directive, and medication outcomes.

As displayed in Figure 1, we defined a rolling 12-month exposure window, anchored quarterly when data pulls were done, during which we determined eligibility and exposure. The exposure period was the 12 months prior to the data pull, and we defined a corresponding pre-exposure period, consisting of the 12 months prior to the beginning of the exposure period. Eligible GeriPACT patients were those with at least 2 PACT visits in the pre-exposure period and at least 2 GeriPACT visits in the exposure period. Eligible PACT patients were those with at least 2 PACT visits in both the pre-exposure and exposure periods. No cross contamination was allowed during exposure period, and no GeriPACT visits were allowed in either group during the pre-exposure period.

### Prospective Matched Cohort Design

Details of the study design and enrollment process were published previously.^10^ Briefly, matching using variables from the pre-exposure period guided prospective enrollment of similar GeriPACT and PACT patients from March 22, 2018, through December 11, 2019. We matched exactly on age within 5 years, EHR-identified race (Black, White, and other [including American Indian or Alaska Native, Asian, Native Hawaiian or Other Pacific Islander, multiracial other than Black, and other selected race]), sex, having 3 or more hospitalizations in the prior year, presence of an advance directive note, dementia diagnosis, and VA site. Among patients matching exactly on these criteria, we selected the best matches without replacement using a Mahalanobis distance function with characteristics—Care Assessment Need scores, JEN Frailty Index (JFI), and number of hospitalizations in the prior year—likely to reflect risk of entry into GeriPACT and high utilization more generally. JFI is the sum of 13 designated EHR frailty categories and estimates long-term care use.^11,12,13^ Care Assessment Need scores, which are expressed as a percentile, reflect how a given patient compares with other VA patients regarding probability of hospitalization or death.^14,15^ We matched 1 GeriPACT patient to 5 or more PACT patients for recruitment with the goal to enroll a match for every enrolled GeriPACT patient. We excluded GeriPACT patients who did not have at least 5 PACT matches. We first attempted to enroll GeriPACT patients and, once a GeriPACT patient was enrolled, attempted to enroll one of their matches. Patient-reported survey data were obtained via telephone interview at the time of enrollment and at 3 subsequent time points (6, 12, and 18 months after enrollment), with the last of these collected June 24, 2021.

### Outcomes

The primary outcome was home time over 18 months starting at the end of the 12-month exposure period (Figure 1 and eMethods in Supplement 1). Home time was selected as an outcome due to its relevance as a surrogate for quality of life and patient independence.^16^ To understand trends in prior periods, we also calculated home time during the pre-exposure and exposure periods to create a longitudinal, 3–time point outcome. Days not at home included those spent in hospital units, short-term nursing facilities, inpatient rehabilitation, or emergency visits; days with outpatient visits or home health, respite, or hospice care were counted as days at home.

Secondary outcomes from EHR data included completion of advance directives (among those without a prior advance directive), screening for falls and incontinence (among those without prior diagnosis of incontinence), and functional assessment, all measured following the first qualifying visit through 24 months afterwards. Potentially inappropriate medications (PIMs) and Anticholinergic Drug Scale (ADS) scores were measured in 6-month intervals around the first qualifying visit, from 12 months prior to the visit through 24 months afterwards. PIMs were coded in each interval as a binary outcome indicating presence or absence, while ADS scores were coded into 3 categories: none, low to moderate (1-2), or high to very high (≥3) anticholinergic activity.

Secondary outcomes from survey data included 2 selected subscales from the validated Patient Perceptions of Integrated Care (PPIC) measure and 5 domains of the Patient-Reported Outcomes Measurement System 29-item Health Profile.^17,18^ The 2 PPIC factors were “provider support for patient’s self-directed care,” which asks patients about the health care professional’s role in understanding their health goals, and “provider support for medication and home health management,” which asks whether health care professionals contacted patients between visits and whether patients had specialist support for medications.^19^ Higher scores indicate higher integration of care.

### Covariates

Covariates were derived from pre-exposure EHR data and survey data, classified into medical, functional, sociodemographic, and economic groupings based on the Andersen model of health care utilization and the International Classification of Functioning, Disability and Health.^20^ Race data were collected to provide information about study participants and the potential generalizability of study results to the overall veteran population.

### Statistical Analysis

Data were analyzed from June 2023 to January 2024. A priori, and based on a randomized clinical trial conducted by our team that used home time,^21^ we estimated that we would have 80% power to detect a 1.5-day difference in home time with sample size of 550 patients (275 dyads).^10^ Standardized mean differences were used to assess balance on covariates across groups for the matched variables and baseline survey variables that were not expected to be impacted by GeriPACT exposure.^22^ Across regressions for all outcomes, facility-level clustering was tested for each outcome via χ^2^ mixture test of random effect variance greater than 0 and was included as needed. Additionally, we adjusted for variables from pre-exposure EHR data as well as survey variables not impacted by GeriPACT exposure. Due to sparseness in some of the screening outcomes, we limited number of covariates included.

Home time was assessed using generalized estimating equations with a log link and negative binomial specification for days not at home with an offset for the number of days an individual was at risk of being not at home (ie, days in the given interval for which they were alive). Empirical SEs were clustered by patient. To estimate the association between GeriPACT and quality-of-care outcomes, we used logistic regression fit with generalized estimating equations and exchangeable working correlation clustered on facility coupled with empirical SEs. For the dichotomized PIMs and Patient-Reported Outcomes Measurement System outcomes, we used logistic regression with individual random effects, including facility-level random effects when indicated, fit with restricted marginal quasi-likelihood estimation to assess differences in GeriPACT vs PACT during each 6-month interval following the first qualifying visit.^23^ For ADS, we used a similar approach but with a proportional odds logistic specification after testing that fit was appropriate, and the facility random effect was omitted due to lack of convergence. PPIC outcomes were fit using linear mixed models with individual and facility level random effects.

To test the robustness of results to inclusion of GeriPACT participants with no enrolled PACT match, we planned an a priori sensitivity analysis for all outcomes by restricting to only matched participants. Analyses were conducted in SAS, version 9.4 (SAS Institute Inc), and tests used a 2-sided *P* < .05 to indicate statistical significance. To aid interpretation, model-estimated outcomes and marginal effects were estimated at the mean values of other covariates in the model.

## Results

### Study Population and Baseline Characteristics

Across 57 VA hospitals that had fully implemented GeriPACT clinics in fiscal year 2016, we identified 3229 eligible GeriPACT patients with at least 5 eligible PACT matches and 40 706 corresponding eligible and matched PACT patients. Matching on patient characteristics and facility, we enrolled 291 GeriPACT patients and 277 well-matched PACT patients (n = 568). For 14 GeriPACT participants, we were unable to enroll a PACT participant match. Median enrollment time between GeriPACT and matched PACT patients was 1 (IQR, 3-11) week. The study flow diagram is presented in the eFigure in Supplement 1.

Mean (SD) age of enrolled participants ranged from 79.7 (6.9) to 81.3 (7.0) years (overall, 80.5 [7.0] years). A total of 10 participants (1.8%) were female and 558 (98.2%) were male. Among the 567 participants with available data, 72 (12.7%) identified as Black; 456 (80.4%), as White; and 39 (6.9%), as other race. Twenty-two of 565 participants with available data (3.9%) identified as Hispanic or Latino. Most participants were married, and most had moderate functional disability (mean [SD] JFI score, 3.9 [1.9]; mean [SD] number of activities of daily living [ADL] dependencies, 1.2 [1.6]).^14,24,25^ Standardized mean differences were less than 0.20 among most baseline variables, indicating that the groups were similar (Table 1).^26^ GeriPACT patients were older, more likely to have cognitive and ADL and/or instrumental ADL impairment, and less likely to have financial security, but these differences were small. In the GeriPACT group, 274 patients (94.2%) had at least 1 subsequent GeriPACT visit in the 18-month postexposure period and 271 (97.8%) of PACT patients had at least 1 additional PACT visit. In aggregate, over the postexposure period, GeriPACT patients had a mean (SD) of 6.5 (5.2) GeriPACT visits (median, 5 [IQR, 3-9]) while PACT patients had a mean (SD) of 7.4 (5.9) PACT visits (median, 6 [IQR, 4-9]).

**Table 1.** Baseline Characteristics by Group

Patient characteristic	Patient group		Standardized mean difference
	GeriPACT (n = 291)	PACT (n = 277)	
Age, mean (SD), y	81.3 (7.0)	79.7 (6.9)	0.22
Race, No. (%)^a^			
Black or African American	36 (12.4)	36 (13.0)	−0.02
White	231 (79.7)	225 (81.2)	−0.04
Other^b^	23 (7.9)	16 (5.8)	0.09
Hispanic or Latino ethnicity, No. (%)^c^	13 (4.5)	9 (3.3)	0.06
Sex, No. (%)			
Female	5 (1.7)	5 (1.8)	−0.01
Male	286 (98.3)	272 (98.2)	0.01
Married, No. (%)	167 (57.4)	169 (61.0)	−0.07
Highest level of education high school or less, No. (%)^d^	89 (30.7)	79 (28.6)	0.05
Advance directive completed, No. (%)	75 (25.8)	63 (22.7)	0.07
Any hospitalizations in prior year, No. (%)	24 (8.2)	21 (7.6)	0.02
Service connected, No. (%)	114 (39.2)	131 (47.3)	−0.16
JFI score, mean (SD)^e^	4.0 (2.1)	3.9 (1.8)	0.05
CAN score, mean (SD), probability percentile	49.5 (32.5)	53.0 (29.5)	−0.11
Dementia or cognitive impairment diagnosis, No. (%)	40 (13.7)	23 (8.3)	0.17
No. of ADLs with help needed, mean (SD)	1.4 (1.7)	1.0 (1.4)	0.26
No. of IADLs with help needed, mean (SD)	2.3 (2.3)	1.7 (2.0)	0.27
No. of chronic conditions [of 18], mean (SD)	3.1 (2.2)	3.3 (1.9)	−0.10
Good or okay financial security, No. (%)^f^	200 (70.4)	217 (78.6)	−0.19
Inadequate health literacy, No. (%)^g^	75 (28.7)	75 (28.4)	0.01
Lives alone, No. (%)	89 (30.6)	75 (27.1)	0.08
Presence of family caregiver, No. (%)^h^	166 (57.2)	161 (58.1)	−0.02
Aid and attendance benefit, No. (%)	10 (3.4)	4 (1.4)	0.13

Abbreviations: ADLs, activities of daily living; CAN, Care Assessment Need; GeriPACT, patient-aligned care team (PACT) with geriatrics-focused care; IADLs, instrumental ADL; JFI, JEN Frailty Index.

^a^ One patient in the GeriPACT group was missing data. Race was self-reported on surveys.

^b^ Includes American Indian or Alaska Native, Asian, Native Hawaiian or Other Pacific Islander, multiracial (except if also selected Black or African American), or other selected race.

^c^ Two patients in the GeriPACT group and 1 in the PACT group were missing data. Ethnicity was self-reported on surveys. Missing values were combined with not of Hispanic or Latino ethnicity for model adjustment.

^d^ One patient in the GeriPACT group and 1 patient in the PACT group were missing data. Missing values were combined with highest level of education high school or less for model adjustment.

^e^ JFI predicts institutional care and other homebased care and services, and ranges from 0 to 13, with higher scores reflecting higher risk of use.

^f^ Seven patients in the GeriPACT group and 1 in the PACT group were missing data. Missing values were combined with poor financial security in adjustment.

^g^ Thirty patients in the GeriPACT group and 13 in the PACT group were missing data.

^h^ One patient in the GeriPACT group was missing data. Missing value was combined with no presence of family caregiver for adjustment.

### Outcomes

Transitioning care to GeriPACT was not associated with home time (rate ratio [RR] for days not at home, 0.97; 95% CI, 0.62-1.51) over 18 months post exposure. In the postexposure period, estimated mean time not at home was 4.64 (95% CI, 3.35-6.43) days in the GeriPACT and 4.81 (95% CI, 3.54-6.53) days in the PACT groups (eTable 1 in Supplement 1). There was also no statistically significant difference in home time during the pre-exposure (RR, 1.15; 95% CI, 0.71-1.87) and exposure (RR, 1.04; 95% CI, 0.68-1.61) periods.

**Figure 2.**
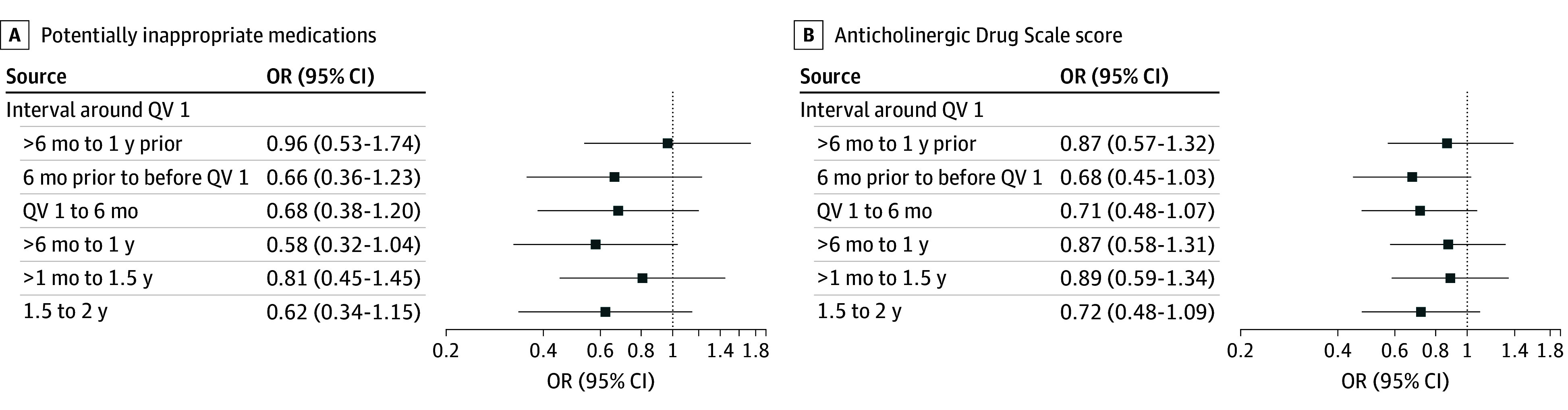
Association of Geriatrics-Focused Care (GeriPACT) With Odds of Presence of Potentially Inappropriate Medications and Anticholinergic Drug Scale Scores The GeriPACT group was compared with the group receiving traditional primary care. OR indicates odds ratio; QV 1, first qualifying visit.

Table 2 and Figure 2 display results of EHR-derived quality-of-care measures. GeriPACT was associated with higher odds of assessment for falls (odds ratio [OR], 2.86; 95% CI, 1.62-5.04), incontinence (OR, 2.73; 95% CI, 1.69-4.42), and function (OR, 4.05; 95% CI, 2.39-6.85). While the increase was not statistically significant, more completed advance directives were observed in GeriPACT patients (OR, 2.11; 95% CI, 0.96-4.62). The estimated probability of a completed advance directive was 0.13 for GeriPACT and 0.07 for PACT. GeriPACT was not associated with differences in odds of a PIM or higher ADS score in any 6-month postexposure outcome window through 24 months (Figure 2 and eTable 2 in Supplement 1). Patient-reported outcomes appear in Table 3. GeriPACT was not associated with higher perceived support for self-directed care, medication management, or patient well-being and self-reported health (Table 3 and eTable 3 in Supplement 1). These results did not change in sensitivity analyses.

**Table 2.** Electronic Health Record Quality of Care Results Within 24 Months After First Qualifying Visit

Outcome measure	No./total No. of patients	OR (95% CI)		
		GeriPACT vs PACT	Probability of screening	
			GeriPACT	PACT
Incontinence screen^a^^,^^b^	468/559	2.73 (1.69-4.42)	0.89 (0.81-0.94)	0.75 (0.64-0.84)
Functional screen^b^	482/568	4.05 (2.39-6.85)	0.92 (0.85-0.96)	0.74 (0.62-0.84)
Falls screen^c^	535/568	2.86 (1.62-5.04)	0.96 (0.93-0.98)	0.89 (0.83-0.94)
Advance directives^a^^,^^c^	44/430	2.11 (0.96-4.62)	0.13 (0.08-0.22)	0.07 (0.04-0.11)

Abbreviations: GeriPACT, patient-aligned care team (PACT) with geriatrics-focused care; OR, odds ratio.

^a^ Individuals with incontinence diagnosis in the 12 months prior to the first qualifying visit or advance directive completed prior to the first qualifying visit were removed from the denominator of the respective models.

^b^ Model covariates include age, economic security, presence of dementia or cognitive impairment diagnosis, cognitive status, marital status, and performance of activities of daily living (ADL) and instrumental ADL. Facility-level clustering was included in estimation of SEs.

^c^ Model covariates include economic security, cognitive status, and ADL performance. Facility-level clustering was included in estimation of SEs.

**Table 3.** Survey-Derived Secondary Outcome Measures of Patient Experience

Outcome (n = 568)	Timing			
	Initial survey	6 mo	12 mo	18 mo
PPIC, mean difference (95% CI)^a^				
Self-directed care^b^	0.05 (−0.11 to 0.22)	0.16 (−0.02 to 0.34)	0.02 (−0.16 to 0.21)	0.12 (−0.07 to 0.32)
PCP support for medication and home health management^c^	0.09 (−0.06 to 0.25)	0.05 (−0.12 to 0.21)	0.14 (−0.03 to 0.31)	0.004 (−0.17 to 0.18)
PROMIS subscale, OR (95% CI)^d^				
Depression^e^	0.93 (0.48 to 1.80)	1.20 (0.59 to 2.46)	1.13 (0.54 to 2.38)	1.48 (0.67 to 3.29)
Pain^f^	0.89 (0.59 to 1.34)	1.17 (0.74 to 1.83)	1.42 (0.88 to 2.31)	1.33 (0.81 to 2.18)
Sleep Disturbance^g^	1.19 (0.61 to 2.35)	1.03 (0.44 to 2.39)	1.21 (0.54 to 2.69)	2.30 (0.99 to 5.31)
Physical Function^h^	1.08 (0.67 to 1.75)	1.27 (0.75 to 2.14)	1.25 (0.73 to 2.13)	1.60 (0.93 to 2.74)
Social Support^i^	0.76 (0.43 to 1.36)	1.43 (0.72 to 2.82)	0.91 (0.46 to 1.79)	1.03 (0.51 to 2.11)

Abbreviations: OR, odds ratio; PCP, primary care practitioner; PPIC, Patient Perceptions of Integrated Care; PROMIS, Patient-Reported Outcomes Measurement System.

^a^ Calculated using linear mixed model with person- and facility-level random effects accounting for repeated measures and facility-level clustering. A positive coefficient indicates a positive association with patient perceptions of integrated care (measured in points). Model covariates include age, economic security, presence of dementia or cognitive impairment diagnosis, cognitive status, marital status, activities of daily living (ADL) and instrumental ADL performance, service connectiveness, ethnicity, educational attainment, presence of a family caregiver, presence of formal aide or attendant, and whether an individual lives alone. Number of observations used in models varied after removing measurements where patient did not complete the survey assessment, was not able to have the outcome scored due to item-level missing data, or, for the self-directed care outcome, the patient volunteered that he/she did not have any primary care appointments in the last 6 months.

^b^ Includes 560 patients at initial survey, 438 at 6 months, 406 at 12 months, and 363 at 18 months.

^c^ Includes 567 patients at initial survey, 474 at 6 months, 446 at 12 months, and 435 at 18 months.

^d^ Calculated using logistic mixed model with person-level random effects. Model covariates include age, economic security, presence of dementia or cognitive impairment diagnosis, cognitive status, marital status, ADL and instrumental ADL performance, service connectiveness, ethnicity, educational attainment, presence of a family caregiver, presence of formal aide or attendant, and whether an individual lives alone. Number of observations used in models varied after removing measurements where patient did not complete the survey assessment or was not able to have the outcome scored due to item-level missing data.

^e^ Facility-level random effects were also included. Includes 560 patients at initial survey, 471 at 6 months, 440 at 12 months, and 431 at 18 months. Modeled as moderate and severe vs within normal limits and mild.

^f^ Modeled as moderate and severe vs within normal limits and mild. Includes 554 patients at initial survey, 465 at 6 months, 426 at 12 months, and 408 at 18 months.

^g^ Modeled as moderate and severe vs within normal limits and mild. Includes 563 patients at initial survey, 468 at 6 months, 442 at 12 months, and 433 at 18 months.

^h^ Modeled as moderate and severe vs within normal limits and mild. Facility-level random effects were also included. Includes 558 patients at initial survey, 464 at 6 months, 432 at 12 months, and 418 at 18 months.

^i^ Modeled as very low and low vs average to very high. Includes 554 patients at initial survey, 458 at 6 months, 425 at 12 months, and 417 at 18 months.

## Discussion

In this cohort study of 568 older adult users of VA primary care, we found that patients who transitioned to GeriPACT had higher rates of recommended screening and assessments. However, there were no differences in home time within 18 months or in self-reported measures of health and well-being or integration of care. The importance of obtaining the patient’s perspective motivated a study design that combined EHR and survey data to generate a comprehensive picture of the effects of geriatrics-focused primary care as implemented in a national health care system. Overall, our findings revealed better care delivery in multiple domains but did not demonstrate immediate improvements in quality of life. Our results are of great relevance to VA, and also outside the VA, as health systems and accountable care organizations look for effective primary care models to improve quality and value for older US residents and enable older adults to remain in the community.

Our study is novel in its examination of the impact of a geriatrics primary care model on home time. Home time has gained appeal as a measure of quality of life because of the preference of many older adults to remain in their homes.^27^ Previous studies examining the impact of primary care models on hospitalizations or emergency department (ED) visits have yielded mixed results.^28,29,30^ In 1 study,^29^ ambulatory intensive care for patients with medically complex needs did not change hospital or ED utilization at 6 months. In a review of studies that targeted elderly high-risk populations, only 1 trial evaluating the Geriatric Resources for Assessment and Care of Elders (GRACE) model found significant reductions in 2-year hospitalizations and ED visits.^30^ It is important to note the usual care comparison condition in the GRACE study and other previously published work; in the GRACE study, participants receiving usual care had low access to services such as social work and case management services; therefore, results may have differed if compared with a model that emphasizes access to team-based care as in the VA’s patient-centered medical home model (PACT).^30^ Indeed, our study revealed that patients in PACT and GeriPACT experienced a mean of 7.4 and 6.5 primary care visits, respectively, over 18 months.

Our secondary outcome results can be framed against a 5-site quality improvement program^31^ that studied the effects of augmenting the VA’s patient-centered medical homes with intensive care coordination. This program had a modestly positive influence on high-risk patients’ experiences with care coordination but did not have a significant impact on most patient-reported access and satisfaction measures.^31^ In the present study we similarly did not observe a difference in patient-reported measures (eg, depressive symptoms, pain, sleep quality). Process of care measures such as recommended screening rates (eg, falls, incontinence, function) were higher in GeriPACT. The ultimate impact of higher screening for geriatric syndromes depends on the prevalence of positive findings and steps taken to mitigate risks for future decline and negative impacts on quality of life; these were not examined in our study. For other survey-based measures of patient experience, we selected patient-centered domains from the PPIC measure, yet we found that GeriPACT patients did not perceive higher integration of self-directed care or medication management.

### Limitations

Our study has limitations. There is a possibility of unobserved confounding as in any nonrandomized evaluation. To minimize risks to validity, our prospective matched cohort design encompassed components of a target trial emulation by exploiting knowledge about the treatment assignment mechanism that drives the joint decision by clinicians and patients to transition to a GeriPACT. We understood the treatment assignment mechanism and matched on factors expected to be associated with treatment and outcomes and used additional controls to minimize bias on the treatment effect, patient preferences for care, and other contextual factors not accounted for. Second, initial patient surveys were conducted after identification of the treatment group, and as such we had no pre-exposure data on patient-reported outcomes to include in models. To minimize unobserved confounding, we controlled for survey baseline variables that were not in a causal pathway. Third, our findings may have been affected by structural differences that were unmeasured (eg, organizational alignment of GeriPACT clinics), and we had no way to validate the involvement of multidisciplinary team members in GeriPACT care. Fourth, we did not adjust for multiple outcomes, preferring to prioritize identifying possible benefit. Despite these limitations, we were able to leverage a large, nationally representative sample of older veterans with detailed longitudinal EHR data coupled with self-reported patient outcomes to include patient perspectives. Information gained can improve understanding of the potential gains and limitations of geriatric-specific primary care as the US grapples with providing optimal care for an aging population. Future work to understand whether there are health care cost differences or lower nursing home entry rates for GeriPACT patients over a 5- to 10-year horizon would also fill gaps in our understanding.

## Conclusions

In this cohort study, geriatrics-focused primary care improved some aspects of quality of care but did not increase home time or other measures of self-reported health and well-being and care integration within the first 18 months of use. Longer-term studies are needed to evaluate whether these observed improvements lead to eventual changes in health such as physical or cognitive function, falls, and health care utilization.

## Notes

Supplement 1. **eMethods.** Additional Details on Study Population and Outcomes **eFigure.** Study Flow Diagram **eTable 1.** Model-Estimated Mean Days Not at Home (95% CI) per Period **eTable 2.** Association of Presence of PIMs and ADS Levels for GeriPACT Patients vs PACT Patients, Odds Ratios and Predicted Probabilities (95% CI) **eTable 3.** Survey-Derived Secondary Outcome Measures of Patient Experience, Estimated Means and Predicted Probabilities (95% CI)
eReferences

Supplement 2.
Data Sharing Statement
