# Supplementary material for: Geriatrics-Focused vs Traditional Primary Care in the Veterans Affairs Health Care System
Source: JAMA Netw Open. 2025 Jan 16;8(1):e2454865. doi: 10.1001/jamanetworkopen.2024.54865 (PMC11739988; doi:10.1001/jamanetworkopen.2024.54865)
Supplement: Supplement 1. — eMethods. Additional Details on Study Population and Outcomes eFigure. Study Flow Diagram eTable 1. Model-Estimated Mean Days Not at Home (95% CI) per Period eTable 2. Association of Presence of PIMs and ADS Levels for GeriPACT Patients vs PACT Patients, Odds Ratios and Predicted Probabilities (95% CI) eTable 3. Survey-Derived Secondary Outcome Measures of Patient Experience, Estimated Means and Predicted Probabilities (95% CI) eReferences [file jamanetwopen-e2454865-s001.pdf]

Supplementary Online Content

Hastings SN, VanHoutven CH, Stanwyck CL, et al. Geriatrics-focused vs traditional primary care in the Veterans Affairs health care system. *JAMA Netw Open*. 2025;8(1):e2454865. doi:10.1001/jamanetworkopen.2024.54865

eMethods. Additional Details on Study Population and Outcomes

eFigure. Study Flow diagram

eTable 1. Model-Estimated Mean Days Not at Home (95% CI) per Period

eTable 2. Association of Presence of PIMs and ADS Levels for GeriPACT Patients vs PACT Patients, Odds Ratios and Predicted Probabilities (95% CI)

eTable 3. Survey-Derived Secondary Outcome Measures of Patient Experience, Estimated Means and Predicted Probabilities (95% CI)

eReferences

This supplementary material has been provided by the authors to give readers additional information about their work.

© 2025 Hastings SN et al. *JAMA Network Open*. This is an open access article distributed under the terms of the CC-BY-NC-ND License, which does not permit alteration or commercial use, including those for text and data mining, AI training, and similar technologies.

eMethods. Additional Details on Study Population and Outcomes

Study Population.
Participants came from sites with fully implemented GeriPACT clinics (500+ annual patients; 800 or fewer patients per team; social worker and pharmacist included on team). Patient-level eligibility criteria were assessed via medical chart review and phone screening. Inclusion criteria were having a valid phone number in the VA medical record and being age ≥65 years. Exclusion criteria included: no phone access or inability to communicate via phone due to hearing impairment; active substance use or psychosis; lack of decision-making capacity and no legally authorized representative to serve as proxy respondent; referral for hospice, palliative care, or prognosis of <6 months to live; or currently in institutional care (nursing home or hospital). The purpose of these criteria was twofold: 1) to promote homogeneity in the sample and therefore reduce risk of bias; and 2) to maximize study retention and completeness of data.

Randomized assignment was not possible because VA directives established GeriPACT as a new model of care open to all eligible patients. Instead, the decision to transfer one's care from PACT to GeriPACT is made jointly between patient, provider, and often a care partner. Therefore, we consulted a panel of experts in geriatric care, including study team members, clinicians, and VA program office partners, to construct a comprehensive list of patient and facility characteristics that could influence the relationship between transferring care to GeriPACT and patient outcomes. The extensive information we had on the treatment assignment mechanism was critical to the validity of the approach.

Outcomes
The primary outcome was home time over 18 months starting at the end of the 12-month exposure period. To understand trends in prior periods, we also calculated home time during the pre-exposure and exposure periods to create a longitudinal, 3 timepoint outcome. Cumulative days not at home were summed across days spent in an observation, medical, or surgical unit in a hospital setting, days spent in a facility-based short-term nursing home or inpatient rehabilitation setting, and days with emergency department visits. Days with outpatient visits, home health, respite or hospice care were considered days at home. Summed days not at home were derived from administrative claims data obtained from VA health records, VA-purchased community care and Centers for Medicare and Medicaid Services. Data from Centers for Medicare and Medicaid Services was provisioned in July 2021 and March 2022.

Presence of a new advance directive (among those not having one prior to the first qualifying visit to either GeriPACT or PACT in the exposure period) was ascertained in a chart review, and coded as completed or not, following procedures adapted from the BEACON trial.[1] Functional assessment and falls and incontinence screening were identified via targeted chart review and recorded as binary measures at any time in the 24 months following the 1st qualifying visit. Incontinence screening was limited to the sample who did not already have an incontinence diagnosis prior to the 1st qualifying visit in the exposure period. These frail elderly performance indicators were derived from the evidence-based Assessing Care of Vulnerable Elders program[2], and endorsed by the VHA Office of Analytics and Business Intelligence and Office of Performance Measurement. Potentially inappropriate medications (PIMs) were defined according to the American Geriatrics Society's Beers criteria, which list medications that should generally be avoided in older adults[3] and which have been used extensively in PIM research for the outpatient setting.[3-5] Anticholinergic drug scale (ADS) was used to assess level of anticholinergic burden across all active medications.[6] Both medication outcomes were measured in 6-month intervals around the 1st qualifying visit, from 12 months prior to the visit through 24 months afterwards. PIMs were coded in each interval as a binary outcome indicating presence or absence, while ADS was coded into 3 categories: none, low/moderate (1-2), or high/very high (≥3) anticholinergic activity.

Secondary outcomes from survey data included selected subscales from the patient perceptions of integrated care (PPIC) measure and 5 domains of the Patient-Reported Outcomes Measurement System 29-item Health Profile (PROMIS-29).[7] We focused on 2 domains of care integration based on their patient-centeredness and psychometric analysis demonstrating satisfactory internal consistency, discriminant validity, and goodness of fit.[8] The two psychometrically derived PPIC factors were "provider support for patient's self-directed care" which asks patients about the provider's role in understanding their health goals and "provider support for medication and home health management" which asks whether providers contacted patients between visits and whether patients had specialist support for medications.[9] These factors used a 4-point scale with a higher score indicating higher integration of care on these domains. Measures of health and well-being were drawn from PROMIS-29 and were dichotomized using established cut-offs for depression, pain, sleep dysfunction, and low physical function (i.e., moderate and severe vs. within normal limits and mild) and low social support (i.e., very low and low vs. average to very high). The PROMIS-29 subscales have good evidence of reliability and responsiveness and have been recommended by the Working Group on Health Outcomes for Older Persons with MCC, an expert panel convened by the National Institute on Aging in collaboration with the Agency for Healthcare Research and Quality.[10]

Covariates
EHR covariates included a dementia or cognitive impairment diagnosis, receiving a VA benefit for needing home help (called Aid and Attendance), and service connection, with higher service connection increasing access to VA care. Baseline survey covariates

© 2025 Hastings SN et al. *JAMA Network Open*. This is an open access article distributed under the terms of the CC-BY-NC-ND License, which does not permit alteration or commercial use, including those for text and data mining, AI training, and similar technologies.

included cognitive status (assessed by modified Telephone Instrument Cognitive Status (TICS-m), Activities of Daily Living and Instrumental Activities of Daily Living, health literacy, age in years, ethnicity (Hispanic vs. non-Hispanic), married, and household member count, presence of a family caregiver, and perceived financial security.[11] Following current best practices, matching was not otherwise accounted for in the analyses (i.e., in variance estimation).[12]

© 2025 Hastings SN et al. *JAMA Network Open*. This is an open access article distributed under the terms of the CC-BY-NC-ND License, which does not permit alteration or commercial use, including those for text and data mining, AI training, and similar technologies.

eFigure. Study Flow Diagram

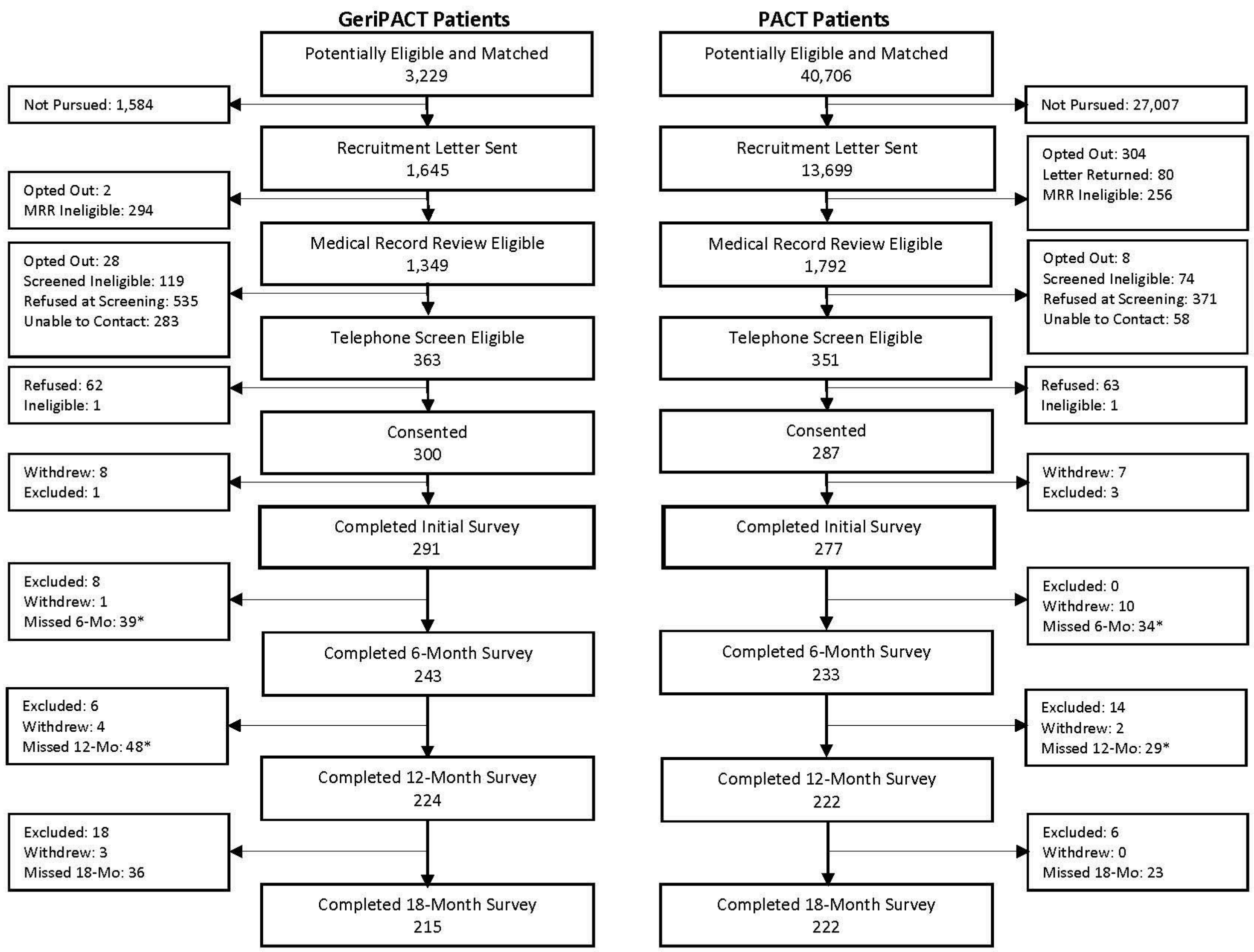

*Missing a survey at one follow-up timepoint does not disqualify a participant from completing a survey at subsequent follow-up timepoints.

This paper analyzes 568 participants, which includes 548 participants of the 550 reported in Smith, et al.[13] Two participants from 1 matched dyad were excluded as the GeriPACT participant was post-hoc identified as having a skilled nursing facility stay covering the entire exposure period. The Smith, et al paper reported only matched dyads; current analyses included 20 additional unmatched participants (either GeriPACT or Pact).

© 2025 Hastings SN et al. *JAMA Network Open*. This is an open access article distributed under the terms of the CC-BY-NC-ND License, which does not permit alteration or commercial use, including those for text and data mining, AI training, and similar technologies.

eTable 1. Model-Estimated Mean Days Not at Home (95% CI) per Period[a]

	Pre-exposure	Exposure	Post-exposure
GeriPACT	2.55 (1.75, 3.63)	3.36 (2.40, 4.69)	4.64 (3.35, 6.43)
PACT	2.19 (1.60, 3.01)	3.22 (2.47, 4.21)	4.81 (3.54, 6.53)

[a] The pre-exposure and exposure periods were each 12 months long, while the post-exposure period was 18 months long. Mean days not at home are estimated at the mean value of all covariates in the model, representing estimates for a "typical" individual.

eTable 2. Association of Presence of PIMs and ADS Levels for GeriPACT Patients vs PACT Patients, Odds Ratios and Predicted Probabilities (95% CI)

	6 to 12 mo. prior to QV1	***6 mo. prior to QV1***	***QV1 and ≤6 mo.***	***>6 and ≤12 mo.***	***>12 and ≤18 mo.***	***>18 and ≤24 mo.***
PIMs (any vs. none)[a], OR (95% CI) n=568	0.96 (0.53, 1.74)	0.66 (0.36, 1.23)	0.68 (0.38, 1.20)	0.58 (0.32, 1.04)	0.81 (0.45, 1.45)	0.62 (0.34, 1.15)
Pr(any PIM fill), GeriPACT (95% CI)	0.12 (0.08, 0.18)	0.09 (0.06, 0.13)	0.12 (0.08, 0.18)	0.11 (0.07, 0.16)	0.12 (0.08, 0.17)	0.09 (0.06, 0.14)
Pr(any PIM fill), PACT (95% CI)	0.12 (0.08, 0.18)	0.13 (0.08, 0.18)	0.17 (0.12, 0.23)	0.17 (0.12, 0.24)	0.14 (0.10, 0.20)	0.14 (0.09, 0.20)
ADS high-very high/low-moderate/none)[a,b], OR (95% CI) n=568	0.87 (0.57, 1.32)	0.68 (0.45, 1.03)	0.71 (0.48, 1.07)	0.87 (0.58, 1.31)	0.89 (0.59, 1.34)	0.72 (0.48, 1.09)
Pr(high-very high ADS), GeriPACT (95% CI)	0.11 (0.08, 0.14)	0.10 (0.08, 0.13)	0.13 (0.10, 0.16)	0.13 (0.10, 0.17)	0.12 (0.09, 0.16)	0.11 (0.08, 0.14)
Pr(high-very high ADS), PACT (95% CI)	0.12 (0.09, 0.16)	0.14 (0.11, 0.18)	0.17 (0.13, 0.21)	0.15 (0.12, 0.19)	0.13 (0.10, 0.17)	0.14 (0.11, 0.18)
Pr(low-moderate ADS), GeriPACT (95% CI)	0.34 (0.28, 0.41)	0.33 (0.26, 0.39)	0.38 (0.32, 0.45)	0.40 (0.34, 0.47)	0.37 (0.31, 0.44)	0.34 (0.28, 0.41)
Pr(low-moderate ADS), PACT (95% CI)	0.37 (0.31, 0.44)	0.42 (0.35, 0.49)	0.47 (0.40, 0.54)	0.44 (0.37, 0.51)	0.40 (0.33, 0.47)	0.41 (0.35, 0.49)

PIMs=potentially inappropriate medications; ADS= anti-cholinergic drug scale; QV1=1st qualifying visit; OR=odds ratio; CI=confidence interval

© 2025 Hastings SN et al. *JAMA Network Open*. This is an open access article distributed under the terms of the CC-BY-NC-ND License, which does not permit alteration or commercial use, including those for text and data mining, AI training, and similar technologies.

[a] Model covariates include age, economic security, presence of dementia/CI diagnosis, cognitive status, marital status, ADL and IADL performance, service connectiveness, ethnicity, education, presence of an informal caregiver, presence of formal aide/attendant, whether an individual lives alone, days of interval follow-up; also included random effects for individual. PIMS model additionally included a random effect for facility, but the ADS model failed to converge with the facility effect included so it was removed. Probabilities are estimated at the mean value of all covariates in the model, representing estimates for a "typical" individual.

[b] Modeling odds of receiving higher ADS category vs. all lower combined (e.g., odds of high-very high ADS vs. low-moderate or none; odds of very high to low vs. none); Assumes proportional odds for each cumulative cut-point.

eTable 3. Survey-Derived Secondary Outcome Measures of Patient Experience, Estimated Means and Predicted Probabilities (95% CI)

	**Initial survey**	**6 month**	**12 month**	**18 month**
PPIC: Self-directed care[a],				
Estimated mean, GeriPACT	2.52 (2.37, 2.67)	2.44 (2.29, 2.60)	2.36 (2.20, 2.52)	2.48 (2.31, 2.64)
Estimated mean, PACT	2.47 (2.31, 2.63)	2.29 (2.12, 2.45)	2.34 (2.17, 2.50)	2.35 (2.18, 2.53)
PPIC: PCP Support for Medication and Home Health Management[a]				
Estimated mean, GeriPACT	2.22 (2.08, 2.37)	2.07 (1.92, 2.22)	2.17 (2.02, 2.33)	2.10 (1.95, 2.26)
Estimated mean, PACT	2.13 (1.98, 2.28)	2.03 (1.87, 2.19)	2.03 (1.87, 2.19)	2.10 (1.94, 2.26)
Promis-29 Depression subscale				
Pr(depressed), GeriPACT	0.09 (0.06, 0.14)	0.10 (0.06, 0.16)	0.10 (0.06, 0.16)	0.09 (0.05, 0.15)
Pr(depressed), PACT	0.09 (0.06, 0.15)	0.09 (0.05, 0.14)	0.09 (0.05, 0.14)	0.06 (0.03, 0.11)
Promis-29 Pain subscale				
Pr(high pain), GeriPACT	0.29 (0.23, 0.36)	0.29 (0.23, 0.37)	0.28 (0.21, 0.36)	0.28 (0.21, 0.36)
Pr(high pain), PACT	0.32 (0.25, 0.39)	0.26 (0.20 0.34)	0.22 (0.16, 0.28)	0.22 (0.17, 0.30)
Promis-29 Sleep Disturbance subscale				
Pr(poor sleep), GeriPACT	0.08 (0.05, 0.12)	0.05 (0.03, 0.09)	0.07 (0.04, 0.12)	0.09 (0.06, 0.15)
Pr(poor sleep), PACT	0.07 (0.04, 0.11)	0.05 (0.03, 0.09)	0.06 (0.03, 0.10)	0.04 (0.02, 0.08)
Promis-29 Physical Function subscale				
Pr(low function), GeriPACT	0.52 (0.43, 0.61)	0.55 (0.46, 0.65)	0.61 (0.51, 0.71)	0.61 (0.51, 0.70)
Pr(low function), PACT	0.50 (0.41, 0.59)	0.49 (0.39, 0.59)	0.56 (0.46, 0.66)	0.50 (0.40, 0.60)
Promis-29 Social Support subscale				
Pr(low support), GeriPACT	0.10 (0.07, 0.15)	0.11 (0.07, 0.16)	0.10 (0.06, 0.15)	0.10 (0.06, 0.15)
Pr(low support), PACT	0.13 (0.09, 0.19)	0.08 (0.05, 0.13)	0.11 (0.07, 0.17)	0.09 (0.06, 0.15)

[a] Linear mixed model with person- and facility-level random effects, a positive coefficient indicates a positive association with patient perceptions of integrated care (measured in points). Model covariates: age, economic security, presence of dementia/CI diagnosis, cognitive status, marital status, ADL and IADL performance, service connectiveness, ethnicity, education, presence of a family caregiver, presence of formal aide/attendant, whether an individual lives alone.

[b] Logistic mixed model with person-level random effects. Model covariates: age, economic security, presence of dementia/CI diagnosis, cognitive status, marital status, ADL and IADL performance, service connectiveness, ethnicity, education, presence of a family caregiver, presence of formal aide/attendant, whether an individual lives alone. Facility-level random effects were also included for depression and physical function models. Depression, pain, sleep dysfunction, and low physical function were modeled as moderate and severe vs. within normal limits and mild. Social support was modeled as very low and low vs. average to very high.

Number of observations used in models after removing measurements where patient did not complete the survey assessment, was not able to have the outcome scored due to item-level missing data, or, for the self-directed care outcome, the patient volunteered that he/she did not have any primary care appointments in the last 6 months.
PPIC Self-directed care: initial=560, 6mo=438, 12mo=406, 18mo=363
PPIC PCP Support for Medication and Home Health Management: initial=567, 6mo=474, 12mo=446, 18mo=435
PROMIS Depression: initial=560, 6mo=471, 12mo=440, 18mo=431
PROMIS Pain: initial=554, 6mo=465, 12mo=426, 18mo=408
PROMIS Sleep: initial=563, 6mo=468, 12mo=442, 18mo=433
PROMIS Physical function: initial=558, 6mo=464, 12mo=432, 18mo=418
PROMIS Social support: initial=554, 6mo=458, 12mo=425, 18mo=417

© 2025 Hastings SN et al. *JAMA Network Open*. This is an open access article distributed under the terms of the CC-BY-NC-ND License, which does not permit alteration or commercial use, including those for text and data mining, AI training, and similar technologies.

eReferences

1. Bailey FA, Williams BR, Woodby LL, et al. Intervention to improve care at life's end in inpatient settings: the BEACON trial. *J Gen Intern Med*. Jun 2014;29(6):836-43. doi:10.1007/s11606-013-2724-6
2. Wenger NS, Shekelle PG. Assessing care of vulnerable elders: ACOVE project overview. *Ann Intern Med*. Oct 16 2001;135(8 Pt 2):642-6. doi:10.7326/0003-4819-135-8_part_2-200110161-00002
3. Stevens MB, Hastings SN, Powers J, et al. Enhancing the Quality of Prescribing Practices for Older Veterans Discharged from the Emergency Department (EQUiPPED): Preliminary Results from Enhancing Quality of Prescribing Practices for Older Veterans Discharged from the Emergency Department, a Novel Multicomponent Interdisciplinary Quality Improvement Initiative. *J Am Geriatr Soc*. May 2015;63(5):1025-9. doi:10.1111/jgs.13404
4. American Geriatrics Society updated Beers Criteria for potentially inappropriate medication use in older adults. *J Am Geriatr Soc*. Apr 2012;60(4):616-31. doi:10.1111/j.1532-5415.2012.03923.x
5. Hastings SN, Schmader KE, Sloane RJ, et al. Quality of pharmacotherapy and outcomes for older veterans discharged from the emergency department. *J Am Geriatr Soc*. May 2008;56(5):875-80. doi:10.1111/j.1532-5415.2008.01648.x
6. Carnahan RM, Lund BC, Perry PJ, Pollock BG, Culp KR. The Anticholinergic Drug Scale as a measure of drug-related anticholinergic burden: associations with serum anticholinergic activity. *J Clin Pharmacol*. Dec 2006;46(12):1481-6. doi:10.1177/0091270006292126
7. PROMIS Score Cut Points. HealthMeasures. Updated 11/3/2023. Accessed May 20, 2024. https://www.healthmeasures.net/score-and-interpret/interpret-scores/promis/promis-score-cut-points
8. Singer SJ, Burgers J, Friedberg M, Rosenthal MB, Leape L, Schneider E. Defining and measuring integrated patient care: promoting the next frontier in health care delivery. *Med Care Res Rev*. Feb 2011;68(1):112-27. doi:10.1177/1077558710371485
9. Ling EJ, Frean M, So J, et al. Differences in patient perceptions of integrated care among black, hispanic, and white Medicare beneficiaries. *Health Serv Res*. Jun 2021;56(3):507-516. doi:10.1111/1475-6773.13637
10. Working Group on Health Outcomes for Older Persons with Multiple Chronic C. Universal health outcome measures for older persons with multiple chronic conditions. *Journal of the American Geriatrics Society*. Dec 2012;60(12):2333-41. doi:10.1111/j.1532-5415.2012.04240.x
11. Michigan Univ. Health and Retirement Study. Ann Arbor, MI: National Institute on Aging; 2012.
12. Stuart EA. Matching methods for casual inference: A review and a look forward. *Stat Sci*. Feb 1 2010;25(1):1-21. doi:10.1214/09-sts313
13. Smith VA, Van Houtven CH, Lindquist JH, Hastings SN. Evaluation of a geriatrics primary care model using prospective matching to guide enrollment. *BMC Med Res Methodol*. Aug 16 2021;21(1):167. doi:10.1186/s12874-021-01360-4

© 2025 Hastings SN et al. *JAMA Network Open*. This is an open access article distributed under the terms of the CC-BY-NC-ND License, which does not permit alteration or commercial use, including those for text and data mining, AI training, and similar technologies.
