# Supplementary material for: Geriatrics-Focused vs Traditional Primary Care in the Veterans Affairs Health Care System
Source: JAMA Netw Open. 2025 Jan 16;8(1):e2454865. doi: 10.1001/jamanetworkopen.2024.54865 (PMC11739988; doi:10.1001/jamanetworkopen.2024.54865)
Supplement: Supplement 2. — Data Sharing Statement [file jamanetwopen-e2454865-s002.pdf]

Data Sharing Statement

Hastings. Geriatrics-Focused vs Traditional Primary Care in the Veterans Affairs Health Care System. *JAMA Netw Open*. Published January 16, 2025.
doi:10.1001/jamanetworkopen.2024.54865

Data
Data available: Yes
Data types: Deidentified participant data
How to access data: susan.hastings@duke.edu
When available: With publication

Supporting Documents
Document types: None

Additional Information
Who can access the data: De-identified data will be made available according to terms of Data Sharing Plan approved by sponsor.
Types of analyses: De-identified data will be made available according to terms of Data Sharing Plan approved by sponsor.
Mechanisms of data availability: De-identified data will be made available according to terms of Data Sharing Plan approved by sponsor.
